# Specific sensorimotor interneuron circuits are sensitive to cerebellar-attention interactions

**DOI:** 10.3389/fnhum.2022.920526

**Published:** 2022-08-19

**Authors:** Jasmine L. Mirdamadi, Sean K. Meehan

**Affiliations:** ^1^Division of Physical Therapy, Department of Rehabilitation Medicine, Emory University School of Medicine, Atlanta, GA, United States; ^2^Department of Kinesiology and Health Sciences, University of Waterloo, Waterloo, ON, Canada

**Keywords:** short latency afferent inhibition (SAI), sensorimotor integration (SMI), cerebellum, theta burst stimulation (TBS), motor, attention, posterior-anterior (PA), anterior-posterior (AP)

## Abstract

**Background:** Short latency afferent inhibition (SAI) provides a method to investigate mechanisms of sensorimotor integration. Cholinergic involvement in the SAI phenomena suggests that SAI may provide a marker of cognitive influence over implicit sensorimotor processes. Consistent with this hypothesis, we previously demonstrated that visual attention load suppresses SAI circuits preferentially recruited by anterior-to-posterior (AP)-, but not posterior-to-anterior (PA)-current induced by transcranial magnetic stimulation. However, cerebellar modulation can also modulate these same AP-sensitive SAI circuits. Yet, the consequences of concurrent cognitive and implicit cerebellar influences over these AP circuits are unknown.

**Objective:** We used cerebellar intermittent theta-burst stimulation (iTBS) to determine whether the cerebellar modulation of sensory to motor projections interacts with the attentional modulation of sensory to motor circuits probed by SAI.

**Methods:** We assessed AP-SAI and PA-SAI during a concurrent visual detection task of varying attention load before and after cerebellar iTBS.

**Results:** Before cerebellar iTBS, a higher visual attention load suppressed AP-SAI, but not PA-SAI, compared to a lower visual attention load. Post-cerebellar iTBS, the pattern of AP-SAI in response to visual attention load, was reversed; a higher visual attention load enhanced AP-SAI compared to a lower visual attention load. Cerebellar iTBS did not affect PA-SAI regardless of visual attention load.

**Conclusion:** These findings suggest that attention and cerebellar networks converge on overlapping AP-sensitive circuitry to influence motor output by controlling the strength of the afferent projections to the motor cortex. This interaction has important implications for understanding the mechanisms of motor performance and learning.

## Introduction

Integrating sensory information from the body and environment is critical to skilled motor performance and learning. Several sensorimotor loops converge on corticospinal neurons in the primary motor cortex to shape motor output. For example, frontoparietal sensorimotor loops may shape afferent projections to the motor cortex to align with various explicit top-down goals and cognitive control processes (Stefan et al., 2004), such as attention. In addition, cerebellar-cortical sensorimotor loops may shape afferent projections to align with implicit cerebellar-mediated processes (Popa et al., 2013). The interaction between the explicit and implicit priorities reflected in the different convergent sensorimotor loops can have beneficial or detrimental impacts on motor cortex physiology, motor performance and learning (Lohse et al., 2014; Suzuki and Meehan, 2020). However, how these sensorimotor loops interact to shape motor output is unclear.

Sensorimotor integration can be probed by measuring the effect of a convergent afferent volley on the motor corticospinal output evoked by transcranial magnetic stimulation (TMS). One such approach is short-latency afferent inhibition (SAI), where a peripheral nerve stimulus inhibits corticospinal motor output. For distal muscles of the upper limb, a median nerve stimulus precedes the TMS stimulus by 18–24 ms so that the sensory afference converges on the motor cortex as the TMS stimulus is delivered (Tokimura et al., 2000). The resulting inhibition is cortical in nature, with the magnitude of SAI determined by the magnitude of the afferent projection to the motor cortex (Bailey et al., 2016). SAI is thought to be mediated by GABAergic and cholinergic mechanisms (Di Lazzaro et al., 2005, 2012). Furthermore, SAI is implicated in motor control processes. For example, SAI is reduced in motor representations involved in an impending behavior (Voller et al., 2006; Asmussen et al., 2014; Suzuki and Meehan, 2020) but enhanced in adjacent motor representations (Voller et al., 2006; Asmussen et al., 2014). Cholinergic involvement in the SAI phenomena also suggests that SAI may provide a marker of cognitive influence over the motor cortex (Suzuki and Meehan, 2020).

Altering the direction of the TMS-induced current used to elicit the motor evoked potential (MEP) in the SAI protocol provides the opportunity to preferentially probe the recruitment of distinct sensory-motor intracortical networks (Ni et al., 2011; Hannah and Rothwell, 2017; Fong et al., 2021). Initially, these distinct interneuron circuits were quantified through the latency of the transsynaptic inputs or “indirect waves (I-waves)” of activity projected onto the corticospinal neuron (Di Lazzaro et al., 1998; Di Lazzaro, 2004; Di Lazzaro and Rothwell, 2014). Compared to the conventional posterior-to-anterior (PA) current, anterior-to-posterior (AP) current is thought to more readily recruit distinct interneuron circuits that give rise to later I-waves, resulting in less synchronized and delayed activity in the corticospinal tract neuron (Day et al., 1989; Di Lazzaro et al., 2001). These physiological differences in PA- vs. AP-circuitry also have different functional consequences. For example, PA-circuitry is linked to model-free learning, while AP-circuitry is linked to cerebellar-mediated, model-based learning (Hamada et al., 2014). Further, cerebellar neuromodulation by anodal transcranial direct current stimulation reduced SAI mediated by interneuron networks recruited by AP, but not PA current (Hannah and Rothwell, 2017).

We recently demonstrated that SAI assessed with AP-, but not PA-induced current decreased during a concurrent visual detection task under high attention load (Mirdamadi et al., 2017). The distinct modulation of AP circuitry by cross-modal attention represents an important mechanism underlying how attention influences sensorimotor processing required for skilled performance and learning. The sensitivity of AP-SAI to both attention (Mirdamadi et al., 2017) and cerebellar modulation (Hannah and Rothwell, 2017) suggests that the AP-SAI interneuron circuit may provide a means for explicit motor control to influence implicit sensorimotor control. However, the consequences of potential interactions between cerebellar-cortical and frontoparietal sensorimotor loops in mediating sensorimotor control processes indexed by SAI are unknown.

The present study investigated this interaction by measuring PA- and AP-SAI during a concurrent visual attention load task before and after cerebellar intermittent theta-burst stimulation (iTBS). Cerebellar iTBS was chosen as cerebellar iTBS reduces the efficacy of plasticity induced by paired-associative stimulation, a protocol that, like SAI, depends on the strength of afferent projections to the motor cortex (Popa et al., 2013). The goal was to determine whether AP-SAI interneuron circuits are a point of convergence for the frontoparietal and cerebellar-cortical sensorimotor loops. To this end, we manipulated frontoparietal activity using a visual detection task with varying perceptual load (Mirdamadi et al., 2017) and cerebellar-cortical activity *via* cerebellar iTBS. We predicted one of two possible outcomes. First, cerebellar iTBS-induced suppression of AP-SAI would summate with attention-induced suppression of AP-SAI, leading to more significant reductions in AP-SAI under higher than low attention load post-cerebellar iTBS. Alternatively, we remained open to the possibility that cerebellar iTBS-induced reductions in AP-SAI combined with attention-induced reductions in AP-SAI would reduce or even reverse the effect of attention load. A reduction or complete reversal of the load effect could arise as a homeostatic response to keep neurons in a functional window in the face of summating suppressive influence. Finally, we have shown that PA SAI is not sensitive to attention load (Mirdamadi et al., 2017), while others have demonstrated that PA SAI is not sensitive to cerebellar modulation (Hannah and Rothwell, 2017). Therefore, we hypothesized that visual attention load would not alter PA-SAI and that cerebellar iTBS would also have no effect on PA-SAI regardless of attention load.

## Materials and Methods

### Participants

Twelve self-reported right-handed adults with no contraindications to TMS participated in the experiment (six males, six females, 22.7 ± 5 years). Participants were screened for contraindications to TMS using a screening questionnaire that included questions about health history and medications (Rossi et al., 2011). All participants reported sleeping at least 6 h the previous evening and eating within the 4 h preceding participation. Participants were excluded if they reported taking medications that could influence the central nervous system. Participants were also screened for color-blindness based upon self-report. All participants were naïve to cerebellar iTBS. However, two participants participated in a prior study during which SAI was assessed concurrently with a similar visual detection task (Mirdamadi et al., 2017). No participants reported adverse responses to either SAI or cerebellar iTBS.

All participants provided written informed consent. The Institutional Review Board of the University of Michigan Medical School (IRBMED) approved the study protocol.

### Experimental design and procedure

**Figure 1:**
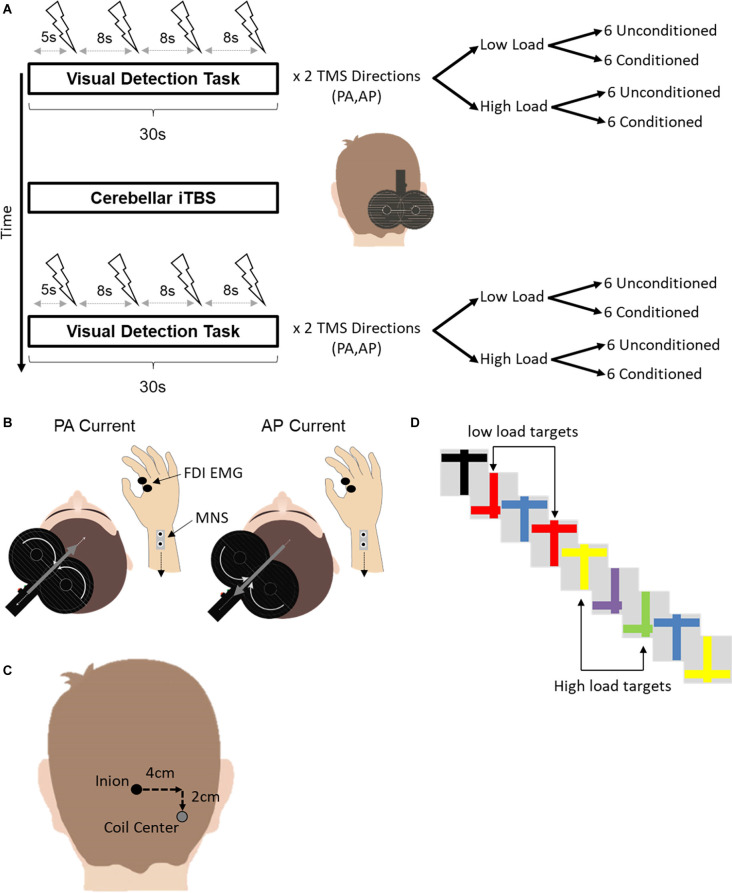
**(A)** Experimental design. Short latency afferent inhibition was assessed during a concurrent visual detection task before and after cerebellar iTBS. The visual detection task consisted of two variants, a low and a high attention load. Four TMS pulses, either conditioned with median nerve stimulation or unconditioned, were delivered over the motor cortex during a single trial of each variant. In total, 24 conditioned and unconditioned MEPs were acquired for each combination of TMS current direction (PA, AP) and attention load (Low Load, High Load) before and after cerebellar iTBS. For conditioned TMS stimuli, electrical stimulation of the median nerve at the contralateral wrist preceded the TMS stimulus by 21 ms. **(B)** A depiction of the different current directions for AP-SAI and PA-SAI. The white curved arrows on the coil indicate the direction of the coil current. The gray arrows indicate the induced current in the brain. Note that the induced current in the brain flows in the opposite direction to the coil current. FDI-EMG, first dorsal interosseous electromyography; MNS, median nerve stimulation. **(C)** An illustration of the TMS coil position for cerebellar iTBS relative to the inion landmark. **(D)** An example of a subset of stimuli used in the visual detection task. In the low load condition (top), participants counted the number of red crosses regardless of orientation. In the high load condition (bottom), participants counted the number of upright yellow or inverted green crosses.

SAI was quantified using motor evoked potentials (MEP) elicited by TMS while participants performed a visual detection task (Mirdamadi et al., 2017) either before cerebellar or after cerebellar iTBS (Figure 1). A single trial of the visual detection task lasted for 30 s, during which a rapid series of upright and upside-down colored crosses were presented on a screen. Four motor evoked potentials (MEP) were elicited using TMS as the crosses were presented. The first TMS stimulus occurred ~5 s after the start of the trial. Each subsequent stimulus occurred at an interval of ~8 s.

Trials were grouped by TMS current direction (PA or AP). Within each current type, there were two attention load conditions (Low Load or High Load). Within each attention load, six trials involved unconditioned TMS stimuli. Six additional trials involved TMS stimuli conditioned by electrical stimulation of the contralateral median nerve at the wrist. Since four TMS stimuli were delivered in each trial of the visual detection task, a total of 24 MEPs were collected for each combination of TMS current direction, attention load and TMS stimulus type (unconditioned, conditioned) before and after cerebellar iTBS.

The order of trials was randomized across participants according to TMS current direction, attention load, and the TMS stimulus type. The order of trials was established pre-iTBS and then repeated following iTBS. The first trial following iTBS was started no sooner than 5 min following the end of cerebellar iTBS.

### Visual detection task

We used a previously published visual detection task (Kamke et al., 2012; Mirdamadi et al., 2017). Participants were required to count pre-defined target stimuli from a rapidly presented stream of different color crosses that were either upright or inverted. Crosses were presented at a rate of 4 Hz. For low load trials, the targets were defined by a single unique feature (any red cross). Defining the target by color created a strong separation between the target and distracter stimuli and has been shown to impose minimal demands on attention resources (Schwartz et al., 2005). For high load trials, targets were defined by a specific combination of color and orientation (upright yellow or inverted green crosses). The conjunction of features used to determine the target placed higher demands on attention as the target was embedded with distracters sharing either color or orientation (Schwartz et al., 2005).

Participants verbally reported their count of target stimuli at the end of the trial. Behavioral performance was assessed by the deviation in the participant’s count compared to the actual number of crosses that met the criterion under the specified load pre- and post-cerebellar iTBS.

### Short-latency afferent inhibition (SAI)

SAI was quantified using monophasic TMS stimuli delivered *via* a MagPro X100 with MagOption stimulator (MagVenture Inc., Atlanta, GA) and a figure-8 coil with a 150° angle between each wing (MC-B70). The coil was oriented tangentially to the scalp over the left motor cortex with the handle at 45° to the midline in the posterior lateral orientation. The coil’s position was held constant throughout the experiment. The current direction was controlled using the stimulator’s onboard software. MEPs elicited by TMS were recorded using LabChart 7 software in conjunction with a Dual BioAmp and PowerLab 8/30 acquisition system (AD Instruments, Colorado Springs, CO).

Participants were seated with both arms on a pillow on their lap. Surface electromyography (EMG) electrodes (Ag-AgCl) were placed over the right first dorsal interosseous (FDI) muscle using a tendon-belly montage. EMG recording was triggered using a 5 V TTL pulse with an epoch of −0.3–0.5 s. During acquisition, data were amplified (×1,000), digitized (×40,000 Hz), and filtered (bandpass filtered 5–1,000 Hz, notch filter—60 Hz). Data were subsequently down-sampled to 5,000 Hz during offline analysis. The MEP was defined as the peak-to-peak amplitude of the maximal electromyographic response between 20 and 50 ms post-TMS stimulation.

The left FDI motor cortical hotspot was defined as the scalp position that elicited the most prominent and consistent MEP in the FDI muscle following PA stimulation. The coil’s location and trajectory on the scalp were recorded using the BrainSight^TM^ stereotactic system (Rogue Research, Montreal, QC). The same hotspot was used for AP stimulation (Sakai et al., 1997). Consistent with our past work (Mirdamadi et al., 2017; Suzuki and Meehan, 2018), test stimulus intensity for SAI was set to the stimulator output that elicited a peak-to-peak MEP amplitude of ~1 mV (in the absence of peripheral stimulation) for each current direction.

SAI consisted of a peripheral electrical stimulus paired with a single monophasic TMS test stimulus. Peripheral electrical stimulation was delivered using a DS7A constant current high voltage stimulator (Digitimer North America LLC, Fort Lauderdale, FL). Stimulation was applied over the median nerve at the right wrist (constant current square wave pulse, 0.2 ms duration, cathode proximal) at an intensity that produced a slight thumb twitch (Abbruzzese et al., 2001). Consistent with our past work (Mirdamadi et al., 2017; Suzuki and Meehan, 2018), electrical stimulation preceded TMS stimulation by 21 ms, an interstimulus interval known to produce the strongest inhibition for PA- (Tokimura et al., 2000; Ni et al., 2011) and AP-SAI (Ni et al., 2011). SAI was derived by expressing the conditioned MEP amplitude as a percentage of the unconditioned MEP amplitude for each current direction and load.

### Intermittent theta burst stimulation (iTBS)

iTBS was delivered using the same MagVenture MagPro X100 with option stimulator. However, the repetitive stimuli were delivered *via* a statically cooled coil (MCF-B70) with the same geometry as the MC-B70 coil used during SAI quantification. iTBS consisted of three biphasic stimuli presented at 50 Hz, repeated every 200 ms for 2 s. The 2 s bursts were repeated every 8 s for a total of 600 stimuli over 190 s. Stimulus intensity was 80% of the FDI’s active motor threshold (AMT; Huang et al., 2005). The active motor threshold was defined as the percentage of stimulator output that elicited an MEP of ≥200 μV peak-to-peak on 10 out of 20 trials during tonic index finger abduction of 20% of the maximum force production (Rossini et al., 2015). These stimulation parameters are consistent with past work demonstrating theta-burst modulation of cerebellar output for at least 30 min (Popa et al., 2010, 2013). iTBS was delivered 4 cm lateral (right) and 2 cm inferior to the inion, previously used to target lobule VIII of the lateral cerebellum, and shown to suppress subsequent paired associative stimulation (Popa et al., 2013). The coil handle pointed superiorly with current induced along the caudal-rostral axis. The vertical handle orientation was previously found to be optimal for inducing measurable behavioral and physiological effects (Théoret et al., 2001; Popa et al., 2013).

### Data analysis

Statistical analyses were performed using the R environment for statistical computing (version 3.6.1; The R Foundation for Statistical Computing). The following packages were used: rstatix (Kassambara, 2020a), “tidyverse” (Wickham et al., 2019), and “ggpubr” (Kassambara, 2020b). All data were normally distributed as assessed by the Shapiro-Wilk test (all *W* > 0.899, all *p* > 0.15).

Visual detection accuracy was assessed using a Current Direction (PA, AP) × Attention Load (Low, High) × Time (pre-iTBS, post-iTBS) repeated measures ANOVA (rmANOVA). The significant three-way interaction was decomposed using separate Attention Load × Time rmANOVAs for each current direction and simple main effects.

SAI analyses were conducted in two steps. First, we evaluated the effect of attention load on SAI before iTBS using a Current Direction (PA, AP) × Attention Load (Low, High) rmANOVA. We also performed a Current Direction (PA, AP) × Attention Load (Low, High) rmANOVA with raw unconditioned MEP amplitude as the dependent variable to detect any differences in unconditioned MEP amplitude. Significant two-way interactions were decomposed using simple main effects.

Second, the modulatory effect of cerebellar iTBS was assessed using a Current Direction (PA, AP) × Attention Load (Low, High) × Time (pre-iTBS, post-iTBS) rmANOVA on SAI. We also followed this analysis with a corresponding Current Direction (PA, AP) × Attention Load (Low, High) × Time (pre-iTBS, post-iTBS) rmANOVA on raw unconditioned MEP amplitude. Significant three-way interactions were decomposed using separate Attention Load × Time rmANOVAs for each Current Direction, followed by simple main effects.

## Results

### Behavioral performance

As expected, behavioral performance was better (i.e., smaller deviation from correct count) in the Low compared to High Attention Load [Main Effect Attention Load: *F*_1,11_ = 13.87, *p* = 0.003; Low = 0.36 ± 0.078 (mean ± standard error); High = 0.75 ± 0.068]. No other significant interactions or main effects for factors of Time or Current Direction were found, suggesting similar deviation pre- and post-iTBS regardless of current direction.

### Short-latency afferent inhibition

**Figure 2:**
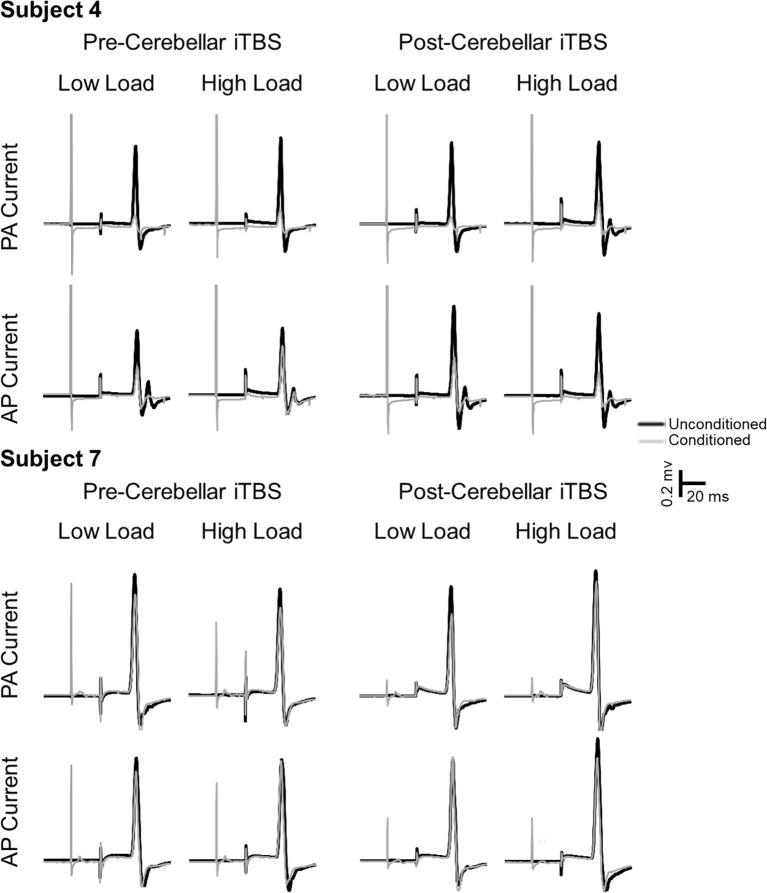
Example MEP traces from two subjects. Black lines represent unconditioned MEPs. Gray lines represent conditioned MEPs. Unconditioned and conditioned traces are grouped by TMS current direction (PA, AP), attention load (low, high) and time relative to cerebellar iTBS (pre, post).

Table 1 lists the individual thresholds for each participant as a percentage of maximal stimulator output. The mean stimulation intensities required to elicit an MEP of 1 mV using PA and AP stimulation were 63 ± 4% and 83 ± 3% of stimulator output. The resting motor threshold for PA and AP stimulation was 50 ± 2% and 70 ± 4% of stimulator output, respectively. AMT was 36 ± 2% of stimulator output. Figure 2 shows individual traces from two participants. Traces illustrate conditioned and unconditioned MEPs for each current direction and attention load before and after cerebellar iTBS.

**Table 1:** Individual TMS thresholds expressed as a percentage of maximal stimulator output.

	**Biphasic**	**PA**		**AP**	
**Subject**	**AMT**	**RMT**	**1 mV**	**RMT**	**1 mV**
					
1	23	45	53	55	70
2	24	41	50	55	74
3	45	61	95	75	87
4	30	47	51	57	75
5	36	47	60	80	89
6	41	64	80	87	95
7	36	45	56	53	63
8	40	44	63	65	92
9	41	54	60	85	92
10	38	52	58	77	87
11	28	47	53	60	74
12	49	57	72	86	95
Average	36	50	63	70	83
SD	8	7	13	13	11

Active motor threshold (AMT) was elicited using the same biphasic stimulus employed by iTBS. The resting motor threshold (RMT) and the threshold to elicit an MEP of 1 mV were defined separately for monophasic posterior-anterior (PA) and monophasic anterior-posterior (AP) current. During SAI assessment, TMS stimulus intensity was set to the stimulator output corresponding to the 1 mV MEP threshold. SD, standard deviation.

A Current Direction × Attention Load × Time rmANOVA on MEP latency confirmed that the latency of the MEP elicited by AP-induced current was significantly longer than the latency of the MEP elicited by PA-induced current regardless of Attention Load or Time (Main Effect_Current_: *F*_1,11_ = 63.58, *p* = 0.00007, η^2^_p_ = 0.85, PA = 21.5 ± 0.2 ms, AP = 23.2 ± 0.2 ms). None of the other effects approached significance.

#### Effect of attention pre-iTBS

**Figure 3:**
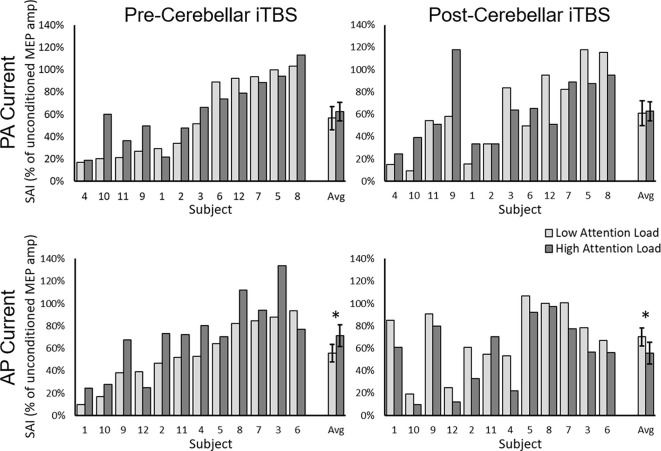
SAI for each participant and the group average (Avg) for PA-current pre-cerebellar iTBS (top left panel), AP-current pre-cerebellar iTBS (bottom left panel), PA-current post-cerebellar iTBS (top right panel) and AP-current post-cerebellar iTBS (bottom right panel). The light bars represent a low visual attention load. The dark bars represent a high visual attention load. Higher values represent lower levels of SAI. Error bars represent the standard error of the mean. ^*^denotes significant group-level comparison (*p* < 0.05). Note, within each current direction, participants are presented from those who demonstrated the greatest SAI to those who demonstrated the least SAI in the pre-cerebellar iTBS low load condition.

The Current Direction × Attention Load rmANOVA upon the pre-iTBS level of SAI revealed a significant Current Direction × Attention Load interaction [*F*_1,11_ = 4.73, *p* = 0.049, η^2^_p_ = 0.30] as well as a significant main effect of Attention Load [*F*_1,11_ = 6.08, *p* = 0.03, η^2^_p_ = 0.36]. The main effect of Current Direction was not significant [*F*_1,11_ = 0.20, *p* = 0.66, η^2^_p_ = 0.02]. Decomposition of the significant interaction using the simple main effect of Current Direction revealed that the interaction was driven by reduced AP-SAI from Low to High Attention Load (*p* = 0.012; Low = 56 ± 8%, High = 72 ± 10%; Figure 3, bottom left panel) but no difference for PA-SAI (*p* = 0.24; Low = 57 ± 10%, High = 63 ± 8%; Figure 3; top left panel).

There were no differences in raw unconditioned MEP amplitude across either current direction or load before iTBS. The Current Direction × Attention Load [*F*_1,11_ = 0.47, *p* = 0.51, η^2^_p_ = 0.04] and main effects of Current Direction [*F*_1,11_ = 0.07, *p* = 0.80, η^2^_p_ = 0.006] and Attention Load [*F*_1,11_ = 0.47, *p* = 0.51, η^2^_p_ = 0.04] all failed to reach significance.

#### Interaction between attention and cerebellar iTBS

The Current Direction × Attention Load × Time rmANOVA on SAI revealed a significant three-way interaction (*F*_1,11_ = 7.04, *p* = 0.022, η^2^_p_ = 0.39; Figure 3) as well as a Time × Attention Load interaction [*F*_1,11_ = 11.58, *p* = 0.006, η^2^_p_ = 0.51]. None of the other effects were significant. Decomposition of the three-way interaction using separate two-way rmANOVAs for each Current Direction revealed a significant Time × Attention Load interaction for AP current [*F*_1,11_ = 19.59, *p* = 0.0009, η^2^_p_ = 0.65]. The interaction was driven by a significant reduction in AP-SAI from Low to High Attention Load pre-iTBS (*p* = 0.011, Low = 56 ± 8%, High = 72 ± 10%; Figure 3, bottom left panel), but an increase in AP-SAI from Low to High Attention Load post-iTBS (*p* = 0.002, Low = 70 ± 8%, High = 56 ± 9%; Figure 3, bottom right panel). None of the effects for the corresponding Time × Attention Load interaction for PA-SAI were significant (Interaction_Time × Attention Load_: *F*_1,11_ = 0.31, *p* = 0.59, η^2^_p_ = 0.03; Main Effect_Time_: *F*_1,11_ = 0.15, *p* = 0.71, η^2^_p_ = 0.01; Main Effect_Attention Load_: *F*_1,11_ = 0.49, *p* = 0.50, η^2^_p_ = 0.04; Figure 3, top left and top right panels).

A Current Direction × Attention Load × Time rmANOVA on unconditioned MEP amplitude suggested that cerebellar iTBS increased unconditioned MEP amplitude [Main Effect Time: *F*_1,11_ = 5.17, *p* = 0.044; Pre = 1,162 ± 87.5 μV, Post = 1,412 ± 81.5 μV]. Importantly, this increase occurred regardless of attention load or current direction, as there were no significant main effects or interactions involving Attention Load or Current Direction.

## Discussion

The current study used cerebellar iTBS and a visual attention load manipulation to determine the interaction between cerebellar and attentional influences on distinct sensorimotor circuits in the motor cortex. Consistent with our past work (Mirdamadi et al., 2017), AP-SAI, but not PA-SAI, was reduced by increasing the attentional demand of a concurrent visual detection task. The novel finding is that cerebellar iTBS did not enhance the attention-related reduction in SAI. Instead, cerebellar iTBS reversed the effect of attentional load on AP-SAI. Following cerebellar iTBS, AP-SAI was enhanced as attentional demand of the concurrent visual task increased. The effects of visual attention load and cerebellar iTBS were specific to AP-SAI interneuron circuits. Neither visual attention demand nor cerebellar iTBS had any impact on PA-SAI.

### Attentional modulation of AP-SAI before cerebellar iTBS

The significant reduction of AP-SAI, but not PA-SAI, with increasing visual attention load replicate our past work (Mirdamadi et al., 2017) and provides further evidence of the distinct functional roles of the sensorimotor circuits probed by the different current directions (Ni et al., 2011; Hamada et al., 2014; Mirdamadi et al., 2017; Suzuki and Meehan, 2018). In our past work, the visual attention load effect on AP-SAI was mirrored in the frontal N30, but not the parietal N20-P25, somatosensory evoked potential (SEP; Mirdamadi et al., 2017). The frontal N30 generator is located in the precentral gyrus (Desmedt and Cheron, 1981), consistent with the proposed origin of the axon terminals targeted by AP-induced current. In contrast, PA-SAI amplitude correlates highly with the parietal N20-P25 SEP amplitude (Bailey et al., 2016). The generator of the parietal N20 is located in Brodmann Areas 3B/1 of the primary somatosensory cortex (Allison et al., 1991). Primary somatosensory pyramidal neurons project to layers V/VI of the motor cortex, the proposed origin of the synaptic terminals targeted by PA-induced current (Aberra et al., 2020). Therefore, the specificity of AP-SAI to the visual attention load effect is consistent with an influence of perceptual load over frontal rather than parietal afferent projections to the motor cortex.

Is PA-SAI unequivocally insensitive to attention? The current study manipulated attention through perceptual load (Schwartz et al., 2005; Kamke et al., 2012). However, attention can be a bottom-up or top-down process directed overtly or covertly to a specific point in space or time. We have demonstrated that focusing attention on the body during skilled action enhances N20-P25 amplitude (Meehan et al., 2009) and PA-SAI (Suzuki and Meehan, 2018). The N20-P25 and PA-SAI are also similarly influenced by increasing working memory load (Suzuki and Meehan, 2018). Therefore, the sensorimotor loops probed by PA-SAI may reflect a higher-order executive control for action selection rather than perceptual processes. In contrast, the specificity of AP-SAI to the visual detection task in the current study is consistent with a unique, convergent, sensorimotor loop influenced by the perceptual load. SAI also interacts with other intracortical mechanisms associated with motor control, such as short intracortical inhibition (Alle et al., 2009; Udupa et al., 2014). How the different sensorimotor loops interact with other intracortical networks to determine motor output is an open question.

### Cerebellar iTBS modulation of AP vs. PA SAI

The observed specificity of cerebellar iTBS on AP-SAI is consistent with past work using cerebellar anodal transcranial direction current stimulation (tDCS) to modulate cerebellar function. Cerebellar anodal tDCS suppressed AP-SAI (Hannah and Rothwell, 2017) elicited by a short ~30 μs pulse width (Hannah and Rothwell, 2017). Cerebellar anodal transcranial direct current stimulation did not influence AP-SAI elicited with longer current durations (120 μs). Consistent with the current study involving cerebellar iTBS, cerebellar anodal tDCS has repeatedly been shown not to affect PA-SAI elicited using either a conventional monophasic stimulator with a fixed pulse width of ~80 μs (Doeltgen et al., 2016) or monophasic stimuli of a short ~30 μs or longer ~120 μs pulse width delivered through a controllable pulse parameter TMS (cTMS) stimulator (Hannah and Rothwell, 2017). As neither cerebellar iTBS nor cerebellar tDCS influence the amplitude of the parietal SEP components (Popa et al., 2013) that are strongly associated with PA-SAI magnitude (Bailey et al., 2016), it is not surprising that we and others have failed to observe any changes in PA-SAI following cerebellar neuromodulation (Hamada et al., 2012; Di Lorenzo et al., 2013; Doeltgen et al., 2016; Hannah and Rothwell, 2017).

No prior work has investigated the effect of cerebellar iTBS or cerebellar tDCS upon AP-SAI elicited with the fixed pulse widths (~72–80 μs) of conventional TMS stimulators. It is plausible that the fixed pulse widths of conventional TMS stimulators recruit a mixture of interneurons sensitive to shorter and longer durations of induced current. The observed specificity of AP-SAI to cerebellar iTBS could be driven by overlapping recruitment of the same interneuron circuits preferentially recruited by the shorter stimulus durations (Hannah and Rothwell, 2017). Future work utilizing a variable stimulus duration is needed to determine whether visual attention demands specifically influence the same AP-SAI interneurons circuits preferentially recruited by short-duration pulse widths or whether they target multiple, unique AP-SAI networks converging on the corticospinal neuron to shape motor cortex output. Regardless, the reversal of the attention load effect on AP-SAI following cerebellar iTBS highlights the functional significance of interactions between convergent frontoparietal and cerebellar-cortical sensorimotor loops to motor control and learning.

### Reversal of the attention effect on AP-SAI by cerebellar iTBS

Cerebellar iTBS did not just abolish the decrease in AP-SAI initially observed under high attention load before iTBS. Post-iTBS, the impact of attention load on AP-SAI reversed. AP-SAI under low attention load was reduced, while AP-SAI under high attention load was enhanced following cerebellar iTBS. The low load condition imposes minimal demands on the attention resources. The reduction in AP-SAI under low load following cerebellar iTBS is consistent with past work demonstrating that both cerebellar iTBS and anodal tDCS suppress afferent projections to AP-sensorimotor circuits (Popa et al., 2013; Hannah and Rothwell, 2017). The minimal impact of cerebellar iTBS on PA-SAI under low load is also consistent with previous reports. Cerebellar anodal tDCS does not influence PA-SAI (Hannah and Rothwell, 2017). While cerebellar iTBS does not influence the N20-P25 SEP generator (Popa et al., 2013), that is the likely one source of afferent projections to PA sensorimotor circuits in the motor cortex (Aberra et al., 2020).

Interestingly, the cerebellar iTBS mediated reduction in AP-SAI under low load (pre-iTBS = 56 ± 8%; post-iTBS = 70 ± 8%) approximated the attention mediated reduction in AP-SAI pre-cerebellar iTBS (low load = 56 ± 8%; high load = 72 ± 10%). The similar reduction indicates that alone, both attention and cerebellar iTBS in isolation suppressed afferent projections to AP-SAI circuits. The subsequent enhancement of AP-SAI with increasing attention load after cerebellar iTBS is consistent with a theoretical homeostatic response to the summation of cerebellar and attention-related suppression under high attention loads following cerebellar iTBS (Ziemann and Siebner, 2008).

Homeostatic mechanisms exist to stabilize neuronal networks and sustain function. The summation of independent attention and cerebellar-mediated suppression of afferent projections to the AP-SAI circuit could theoretically have substantially decreased the excitability of and destabilized these sensorimotor circuits. In the face of strong cumulative suppression, a homeostatic mechanism would increase the neuron’s gain to maintain a functional response and stabilize the sensorimotor circuit leading to enhanced levels of SAI. Consistent with this theory, the combined cerebellar and attention mediated effects on AP-SAI seen under high load after cerebellar iTBS (56 ± 9%) approximated the magnitude of AP-SAI observed with minimal cerebellar and attention influence seen under low load before cerebellar iTBS (56 ± 8%).

An alternate explanation for the reversal of SAI by cerebellar iTBS is that the locus of the cerebellar iTBS was not exclusive to motor areas of the cerebellum. The cerebellar volume targeted (lobule VIII) has sensorimotor and cognitive functions (Bushara et al., 2001; Stoodley and Schmahmann, 2009). The cerebellum has widespread connections with many cortical areas, including the prefrontal cortex. Therefore, we cannot rule out that our iTBS conditioning changed cerebellar-prefrontal connectivity leading to altered attentional control (Schmahmann, 1991, 2019; Strick et al., 2009; Rastogi et al., 2017). However, a breakdown of prefrontal attention control seems less likely. Prefrontal cortex lesions reduce the ability to suppress task-irrelevant somatosensory afference to both frontal and parietal SEP generators (Yamaguchi and Knight, 1990). Given the strong association between PA-SAI and the activity of the parietal SEP generators (Bailey et al., 2016) any generalized breakdown of prefrontal attention control following cerebellar iTBS should have influenced both AP-SAI and PA-SAI. The cerebellar-prefrontal connectivity account is also mitigated by our previous demonstration that the visual attention load manipulation employed here selectively influenced the frontal P20-N30, but not the parietal N20-P25, SEP component (Mirdamadi et al., 2017). Finally, there was no change in visual task accuracy following cerebellar iTBS, suggesting that participants maintained visual task performance. Therefore, it is more likely that the reversal of SAI reflects a homeostatic response to the summated suppression associated with cerebellar iTBS and an increasing attention load rather than compromising the executive control of attention.

### Limitations

The current study has several important limitations that should be considered. First, we did not include a sham cerebellar iTBS condition or alternative stimulation site to control for non-specific effects of TBS. Instead, we employed different current directions and visual attention loads to control non-specific effects within a session. We did observe a general increase in unconditioned MEP amplitude following cerebellar iTBS, regardless of current direction and visual attention load. However, the effect of cerebellar stimulation was specific to AP-SAI, and the pattern of change in AP-SAI from low to high visual attention load was in the opposite direction pre and post-cerebellar iTBS. Therefore, a generalized effect is unlikely. That said, future work should employ a continuous theta burst (cTBS) cerebellar condition. Establishing the opposite or an inert effect following cTBS cerebellar stimulation would provide the most robust control to rule out non-specific effects of iTBS under similar sensory conditions.

A second limitation is that our visual detection task makes the correlation between SAI changes and task performance challenging to quantify. The high load condition in the visual detection task yielded significantly poorer performance. However, there was very little variation in accuracy across participants. The absence of substantial variance in accuracy likely reflects that load in our accuracy measure is a combination of two outcomes, including false-positive identification of targets and the omission of missed targets in the reported count. In contrast, a metric like response time would more consistently capture variability in attention load. Unfortunately, we were not able to quantify response time. Reporting a count at the end of the trial reduced interactions between attention load, SAI and the motor response that would have been present if participants were required to press a response key. A verbal response during the task was also not possible as it would induce head movement and decrease the precision of the TMS localization.

A third limitation is that we could not establish the time course of the effect of cerebellar iTBS. The effects of iTBS have a definite period, with the strength decaying over time. Due to the need to collect sufficient unconditioned and conditioned trials under each combination of current direction (PA, AP) and visual attention load (Low, High), we could not collect multiple time points within the critical window. Given the specificity of cerebellar iTBS on AP-SAI, future work should establish the time course of the interaction between cerebellar iTBS and visual attention load in the AP-SAI circuit.

A final limitation is that we did not adjust the test stimulus intensity required to induce an MEP of ~1 mV following cerebellar iTBS. As noted, we did observe a general increase in unconditioned test stimulus MEP amplitude following cerebellar iTBS. However, the general increase in unconditioned test stimulus amplitude was consistent across both current directions and attention loads. The specificity of the cerebellar SAI effects on AP-SAI under a high attention load suggests that any global increase in cortical excitability cannot explain the reversal of AP-SAI post-cerebellar iTBS.

## Conclusion

Consistent with our past work, increasing visual attention load suppressed SAI evoked with AP current but not PA current. This preferential change in AP-sensitive circuitry was also observed for the effects of cerebellar iTBS on SAI. The differential effects of cerebellar iTBS on SAI for low vs. high attention load suggest that cerebellar and attentional mechanisms converge and alter sensory afferents onto AP-mediated motor cortical circuitry. The interaction between attention and cerebellar effects upon AP SAI networks may be a substrate by which explicit sensorimotor control mechanisms shape implicit, procedural sensorimotor processes in the motor cortex.

## Data Availability Statement

The raw data supporting the conclusions of this article will be made available by the authors, without undue reservation.

## Ethics Statement

The studies involving human participants were reviewed and approved by Institutional Review Board of the University of Michigan Medical School (IRBMED). The patients/participants provided their written informed consent to participate in this study.

## Author Contributions

SM and JM contributed to the conception and design of the study. SM performed the statistical analyses. JM wrote the first draft of the manuscript. All authors contributed to the article and approved the submitted version.

## Funding

The present work was partially supported by research funding from the Natural Sciences and Engineering Research Council of Canada (NSERC; RGPIN-2020-04255) and a KL2 Scholar award from the Claude D. Pepper Older Americans Independence Center at the University of Michigan to SM (P30AG024824).
